# Effects of prebiotics, probiotics, and synbiotics on the prevention and treatment of cervical cancer: Mexican consensus and recommendations

**DOI:** 10.3389/fonc.2024.1383258

**Published:** 2024-03-28

**Authors:** Gabriela Gutiérrez Salmeán, Merari Delgadillo González, Ariadna Alejandra Rueda Escalona, José Antonio Leyva Islas, Denisse Castro-Eguiluz

**Affiliations:** ^1^ Centro de Investigación en Ciencias de la Salud (CICSA), Facultad de Ciencias de la Salud, Universidad Anáhuac Mexico, Huixquilucan, Estado de Mexico, Mexico; ^2^ Servicio de Nutrición, Centro de Especialidades del Riñón (CER), Naucalpan de Juarez, Estado de Mexico, Mexico; ^3^ Modelo Integral para la atención del Cáncer Cervicouterino Localmente Avanzado y Avanzado (MICAELA) Program, Instituto Nacional de Cancerología, Mexico City, Mexico; ^4^ Masters in Medical Sciences Program, Universidad Anáhuac Mexico, Huixquilucan, Estado de Mexico, Mexico; ^5^ Nutritional and Metabolic Support, Instituto de Seguridad y Servicios Sociales de los Trabajadores del Estado (ISSSTE) Hospital Regional Lic. Adolfo López Mateos, Mexico City, Mexico; ^6^ Investigador por México, Consejo Nacional de Humanidades Ciencias y Tecnologías (CONAHCyT)—Department of Clinical Research, Instituto Nacional de Cancerología, Mexico City, Mexico

**Keywords:** human papillomavirus, HPV-infection, cervical cancer, chemoradiotherapy, prebiotic, probiotic, synbiotic

## Abstract

Gut microbiota plays a crucial role in modulating immune responses, including effector response to infection and surveillance of tumors. This article summarizes the current scientific evidence on the effects of supplementation with prebiotics, probiotics, and synbiotics on high-risk human papillomavirus (HPV) infections, precancerous lesions, and various stages of cervical cancer development and treatment while also examining the underlying molecular pathways involved. Our findings indicate that a higher dietary fiber intake is associated with a reduced risk of HPV infection, while certain probiotics have shown promising results in clearing HPV-related lesions. Additionally, certain strains of probiotics, prebiotics such as inulin and fructo-oligosaccharides, and synbiotics decrease the frequency of gastrointestinal adverse effects in cervical cancer patients. These agents attain their results by modulating crucial metabolic pathways, including the reduction of inflammation and oxidative stress, promoting apoptosis, inhibiting cell proliferation, and suppressing the activity of oncogenes, thus attenuating tumorigenesis. We conclude that although further human studies are necessary, robust evidence in preclinical models demonstrates that prebiotics, probiotics, and synbiotics play an essential role in cervical cancer, from infection to carcinogenesis and its medical treatment. Consequently, we strongly recommend conducting high-quality clinical trials using these agents as adjuvants since they have proven safe.

## Introduction

Cervical cancer (CC) ranks fourth among all cancers in women worldwide (1). CC is primarily caused by persistent infection with high-risk human papillomavirus (hrHPV) strains (2). hrHPV infection causes abnormal changes in the cervix epithelium, which may lead to non-oncogenic (benign) or precancerous lesions that can further progress to cancerous growths (3, 4).

As mucosal surfaces are highly exposed to potentially harmful microorganisms, the mucosal-associated immune response plays a crucial part in the pathologic course of infections. In this context, the role of microbiota in vaginal health has been extensively studied. A low diversity of organisms characterizes the vaginal mucosa, being the Lactobacillus species the most abundant within the vaginal microbiota. Lactobacilli produce lactic acid and short-chain fatty acids (SCFAs) that acidify the microenvironment and prevent the establishment of pathogenic organisms. Vaginal epithelial cells produce adequate mucus that protects the mucosal barrier helping maintain an anti-inflammatory environment. An increase in pH, dysbiosis, and inflammation disrupt the vaginal mucosal barrier, which promotes the generation of reactive oxygen species (ROS), genomic instability, and, consequently, a pro-tumorigenic environment. In light of the importance of the microbiome in vaginal health, interventions that maintain and restore the microbiota are warranted.

Gut microbiota influences the body’s response to pathogens, including viral infections (5). Studies have demonstrated that certain probiotic strains can inhibit the growth of hrHPV, decrease viral load, and promote effector immune responses against the infection by enhancing the activity of immune cells, including natural killer cells and T cells, which play crucial roles in fighting cancer (6–11). Thus, maintaining a balanced gut microbiome (eubiosis) may enhance the immune response against hrHPV and potentially reduce the risk of CC or improve antineoplastic response (4). In this context, modulating the microbiota poses an opportunity in several stages of the disease process of CC.

*Probiotics* are live microorganisms that confer health benefits when consumed adequately in both quantity and quality, and their consumption may enrich the microbiota (12). *Prebiotics* are defined as substrates selectively used by probiotic microorganisms, which promote their establishment, growth, and diversity in the gut. Increased gut microbiota diversity is associated with a reduced risk of several cancers, including CC (13). The prebiotic agents more frequently considered are non-digestible fibers (e.g., oligomers from glucose and fructose); however, emerging evidence has also turned attention to other substrates, including resistant starches, some vitamins, flavonoids and polyphenols, human milk, and many other components found in functional foods (14, 15). *Synbiotics* are a mixture of probiotics and prebiotics aimed to potentiate the health benefits to the host (13). These regulate gut microbiota composition and function, and as the microbiota participates in immunomodulatory processes, synbiotics may hold promise in preventing and treating CC (16). Moreover, synbiotics have been shown to attenuate the severity of cancer treatments’ side effects—as both chemotherapy and radiation therapy can disrupt the gut microbiota and frequently cause diarrhea and intestinal inflammation— (17–19).

Finally, *postbiotics* are defined as preparations of inanimate microorganisms and their metabolites, which provide the biological mechanism associated with health benefits to the host (20). Studies *in vitro* have shown that postbiotics can exert cytotoxicity, arrest proliferation, and promote apoptosis in cancer cells while activating protective and stress-resistant mechanisms in healthy cells (18).

The mechanisms through which prebiotics, probiotics, synbiotics, and postbiotics could modulate the immune response in hrHPV-infected women, prevent CC in patients with premalignant lesions, or benefit CC patients during treatment are not fully understood. However, gut microbiota balance, immune modulation, and enhanced antiviral responses are likely involved (21). As part of the 2023 Mexican National Consensus in Cervical Cancer, we investigated the effects of supplementation with prebiotics, probiotics, synbiotics, and derived metabolites on CC. Here, we present the results of the investigation and the consensus-derived recommendations.

## Methods

We conducted an evidence-based review of the interaction and the effect of prebiotics, probiotics, synbiotics, and postbiotics in hrHPV infection, precancerous lesions, and different stages of CC.

The literature search was performed in Pubmed, Web of Science, and Cochrane Library databases. The results included clinical and experimental studies, clinical trials, and observational studies published in English without restriction on publication year. Key search terms combine disease, type of treatment, and intervention terms. The search terms for disease included “human papillomavirus infection,” “cervical intraepithelial neoplasia (CIN),” and “cervical cancer.” The search terms for treatment included “surgery,” “chemotherapy,” “chemoradiotherapy,” and “palliative care.” The search terms for intervention included “probiotics,” “prebiotics,” “synbiotics,” “probiotics metabolites,” and “dietary fiber.”

## Results and discussion

Most of the evidence found in the literature on interventions with prebiotics, probiotics, or synbiotics focused on managing diarrhea and other gastrointestinal toxicity symptoms in CC treatment. Few studies focused on CC prevention, and even fewer on preventing infection with HPV or vaccine efficacy. We discuss the evidence according to the natural evolution of the disease.

### Prevention and clearance of HPV infection

Vaginal microbiota may play a role in the risk of hrHPV infection and the development of CC (22). The presence of Lactobacillus has been shown to protect the vaginal mucosa and reduce the risk of HPV infection (23). Infection with HPV changes the microbial composition of the vaginal microbiota; these changes may be involved in CC progression. In a study by Santella et al., Actinobacteria, Proteobacteria, and Bacteroides were more represented in HPV+ patients (5). Studies have also shown that Lactobacillus can promote vaginal epithelium tissue repair and clearance of HPV infection. Mainly, *L. rhamnosus* and *L. salivarius* exerted the highest cytotoxic effect on HeLa cells infected with HPV-16 and 18 and produced lactic acid, hydrogen peroxide, bacteriocins, biosurfactants, and exopolysaccharides. Intervention studies with probiotics supplementation have shown dissimilar results. Palma et al. studied the effect of long-term vaginal application of *Lactobacillus rhamnosus* BMX 54 (10^4^ CFUs) for short-term (3 months) or long-term (6 months) periods to restore vaginal ecosystem in HPV infection. The long-term group had a better chance of resolving HPV cytological anomalies (79.4% vs 37.5%, p=0.04). HPV clearance was 31.2% in the long-term group, compared to 11.6% in the short-term group (p=0.04) (24). *Lactobacillus crispatus* M247 is more frequent in non-infected women and was shown to have fecal and vaginal colonizing properties; according to a study by Dellino et al., oral administration with *Lactobacillus crispatus* was investigated in HPV-infected women. HPV clearance was 9.3% in controls vs. 15.3% in the probiotic group (8). Another study assessed the influence of probiotics on genital hrHPV clearance. Oral supplementation with probiotics *Lactobacillus rhamnosus* GR-1 and *Lactobacillus reuteri* RC-14 (5.4 billion CFUs) showed no differences in clearance compared to placebo groups (7). In women with HPV+ precancerous lesions, a 6-month intervention with a daily probiotic drink (*Lactobacillus casei Shirota*) showed a clearance rate of 60% in the probiotic vs. 31% in the placebo group (p=0.05) (9). Because intestinal and vaginal microbiota are linked, oral supplementation with probiotics seems a promising approach to achieve eubiosis in women infected with HPV.

Regarding prebiotics, in a Nahnes study among 14151 women, intake of dietary fiber was negatively associated with HPV infection. An increase in dietary fiber was associated with a lower risk of HPV infection (OR, 0.43; 95% CI 0.38-0.48) (25).

Among other reported non-invasive therapies to clear HPV infection, herbal treatments that include phenolic compounds helped clear the infection (26). An exciting approach by Seo et al. assessed the combined effect of diet and cervical microbiome on the risk of CIN. Association between the semi-Western diet (lower intake of vegetables, fiber, carotene, vitamin C, and Se), in addition to the abundance of *A. vaginae*, increased the risk of CIN; on the other hand, the prudent diet was associated with elimination of CIN (27). We stress the importance of an adequate dietary pattern to favor the establishment of probiotic organisms. The *Milpa diet* is a traditional Mexican food pattern that includes foods consumed since prehispanic times, mainly corn/maize, beans, squash, chili peppers, tomato, cactus pads, cacao, and avocado (28). In observational studies, adherence to this food pattern has shown health benefits that may be attributed to phenolic compounds, saponins, carotenoids, and galacto-oligosaccharides, which act as prebiotics and thus modulate gut microbiota and systemic inflammation (29, 30).

*Our consensus recommendations for prevention and clearance of HPV infection are:*

1. Dietary fiber consumption, according to FAO recommendations.
  - Children: 14 to 25g, beginning at weaning.
  - Adolescents: 26g.
  - Adults: 25g.
2. Emphasize the consumption of soluble fiber (1g per 3g of insoluble fiber) for its prebiotic effect.
3. Include phenolic-rich foods from fruits, vegetables, and spices.
4. Include, since weaning throughout all life stages, the consumption of probiotics through fermented foods.
5. Vaginal application of probiotics *Lactobacillus casei* and *Lactobacillus rhamnosus* (10^4^ CFUs).
6. Oral supplementation of probiotic *Lactobacillus crispatus* (between 10^8^ and 10^10^ CFUs).
7. Habitual consumption of the Milpa diet as a tropicalization of the prudent and Mediterranean diets.

### CIN regression and CC prevention

The dysbiosis environment in the vagina conditions the progression from CIN to CC. Probiotics have been shown to promote regression of CIN (RR 0.48) (26). Postbiotic metabolites, SCFAs, and other antibacterial compounds secreted by Lactobacillus keep a low vaginal pH and protect against the establishment of vaginal pathogens associated with CIN and CC. For example, linoleic acid produced by such bacteria modulates the gene expression of cellular response to growth factors, thus affecting cell proliferation, survival, and differentiation (31). The supernatant of *L. fermentum* from healthy controls exhibited probiotic characteristics and antibacterial activities against some pathogens, including organisms isolated from CIN and CC patients (32). A study *in vitro* demonstrated that *L. plantarum* and *L. acidophilus* not only inhibit the establishment of pathogenic bacteria preventing vaginal dysbiosis and increased CIN but also secreted postbiotic compounds inhibit and prevent the growth of malignant cells and cancer progression (33).

A diet rich in plant-based nutrients may reduce the risk of CC. Significant risk reductions of 40-60% were observed for women in the highest vs. lowest tertile for dietary fiber, vitamins C, E, A, alfa- and beta-carotene, lutein, folate, and total fruit and vegetable intake (34). This study supports the recommendation of a diet rich in fruits and vegetables for preventing progression to CC in patients with CIN. The World Health Organization (WHO) and Food and Agriculture Organization (FAO) recommend a daily intake of 5 portions of fruits and vegetables. Interestingly, low-income and least-developed countries do not reach the recommendation (35, 36). Healthcare providers and public health institutions should emphasize increasing the consumption of fruits and vegetables.

The protection fruits and vegetables provide derives from the antioxidant capacity and immune-modulating effect of phenolic compounds and metabolites from fiber fermentation.

*Our consensus recommendation for CIN regression and CC prevention are:*

1. Consumption of at least 200 g of fruit and 200 g of vegetables daily.
2. Consumption of probiotics from fermented foods or supplementation with *Lactobacillus rhamnosus* and *Lactobacillus crispatus* (between 10^8^ and 10^10^ CFUs).

### Antitumoral effect on CC and safety in CC patients

The oncoprotective effects of lactobacillus metabolites and SCFAs in CC were demonstrated in studies *in vitro* and animal models. SCFAs exert a cytotoxic effect on HeLa cells in a concentration-dependent manner (37). Several mechanisms of action of probiotics have been identified to inhibit cancer cells and enhance antitumoral immunity, including Th1 response, NK cell activation, cytotoxic T cell activation, increased dendritic cell-antigen presentation, and increased production of IgA (38–40).

Studies have reported concern about the risk of supplementation with probiotics in the development of adverse events in CC patients, such as infection, septicemia, lactose intolerance, stomach pain, and bloating. A systematic review and meta-analysis assessed the safety of probiotics in CC patients (41). Patients in the probiotic groups experienced less frequency and severity of diarrhea than the placebo group (RR 0.61; 95%CI 0.46-0.81, p<0.0001). The adverse events (fever, anorexia, stomach pain, and bloating) were not different among groups. Evidence suggests a protective role of probiotic supplementation, associated with an anti-inflammatory effect. The mechanisms could involve competition with other pathogenic organisms, immune modulation of the inflammatory response, and the production of mucus that protects the integrity of the epithelial barrier (42).

*Our consensus recommendation for antitumoral effect and CC safety are:*

1. We recommend both oral consumption and vaginal application of Lactobacillus rhamnosus, Lactobacillus reuteri, Lactobacillus plantarum, Lactobacillus acidophilus, Lactobacillus crispatus, and Lactobacillus gasseri, at least 10^4^ CFUs through vaginal application, and between 10^8^ and 10^10^ CFUs orally.
2. Regarding the long-held concern that live probiotic microorganisms may cause infection, even sepsis, in immunocompromised cancer patients, we believe that live probiotics are safe and promote adequate immune responses, including tolerance to synbiotic organisms.

### Effect on radiotherapy-derived symptom management in locally advanced CC patients during concurrent chemoradiotherapy

The use of probiotics in cancer patients during oncological treatment has been questioned. Risk of septicemia, bacteremia, and other adverse effects unrelated to radiotherapy (RT) treatment have been traditionally, without scientific evidence, attributed to probiotic supplementation. However, we seek to prove that no evidence demonstrates a risk for adverse effects with probiotic consumption. In fact, in probiotic interventions investigated during concurrent chemoradiotherapy (CRT), most adverse effects reported are not different from those secondary to CRT, and probiotic supplementation may confer some protection, particularly from the development of diarrhea. Some possible mechanisms include that beneficial bacteria and their metabolites induce mucus production within the intestinal wall and restore the vascular and connective tissues damaged by RT (43, 44).

The effect of probiotics for the prevention of acute RT-induced diarrhea has been investigated in CC patients by Linn et al. A probiotic containing *Lactobacillus acidophilus* LA-5 and *Bifidobacterium animals lactis* (BB-12) (1.7 billion CFUs 3 times a day) was consumed during CRT. Reduced incidence of diarrhea was reported in the probiotic group compared to placebo (53.8 vs 82.1%, p<0.05), and delayed onset of diarrhea and less frequent use of loperamide. Notably, the pain was also less frequent in the probiotic group (45). Delia et al. also investigated the use of probiotics to prevent RT-induced diarrhea in sigmoid, rectal, or CC patients who received adjuvant postoperative RT. The probiotic preparation tested was VSL#3 (one sachet a day from the first day of RT until the end of RT) vs placebo. The probiotic group had less frequency and severity of diarrhea (51.8 vs 31.6%, p<0.001). The VSL#3 probiotic proved safe; no bacteremia, sepsis, or septic shock cases were reported (46). A randomized clinical trial supplemented LACC patients receiving CRT with 10^9^ CFUs of *Lactobacillus acidophilus* and *Bifidobacterium bifidum* twice daily to prevent diarrhea. Reduced incidence of RT-induced diarrhea and the need for anti-diarrheal medication were observed, with benefits on stool consistency (47). The effect of probiotic yogurt with 10^8^ CFUs of *Lactobacillus casei* DN-114 001 was assessed to prevent RT-induced diarrhea in gynecological cancer patients. The probiotic drink significantly impacted stool consistency but not grade >2 diarrhea (48).

Regarding the use of prebiotics, an intervention during RT treatment analyzed the effect of inulin and fructo-oligosaccharide, compared to placebo, on the microbiota of patients. Three weeks after treatment, the prebiotic group had more abundance of Lactobacillus and Bifidobacterium (49). A follow-up study by the same author evaluated the effect of 6g of prebiotic supplementation in preventing acute radiation enteritis in gynecological cancer patients. The frequency of watery stool was lower in the prebiotic group (50). So, prebiotic supplementation may help recover important microbiota species and improve the consistency of stools during RT treatment.

A symbiotic supplementation that consisted of a biogel that contained 10^7^ CFUs of *Lactobacillus acidophilus*, 10^6^ CFUs of *Bifidobacterium lactis*, and blue agave inulin three times daily for seven weeks during CRT resulted in lower fecal calprotectin, better stool consistency, and less frequency and severity of vomit (51). Therefore, an additional benefit in the probiotic intervention results from adding prebiotics to reduce local inflammation and manage other symptoms.

These studies demonstrate that during and after treatment, oral administration of probiotics, mainly lactic acid bacteria, is well tolerated by CC patients and has been proven safe in several clinical trials. The consumption of soluble fiber as a prebiotic ensures the establishment of probiotic organisms and the production of SCFAs.

*Our consensus recommendations for symptom management during CRT are:*

1. Prophylactic use of probiotics *Lactobacillus acidophilus* and *Bifidobacterium bifidum* at a dose of 10^9^ CFUs twice daily, at least seven days before RT begins and up to 3 months after treatment completion.
2. The probiotic supplementation must be given with adequate amounts of dietary fiber and prebiotics (1g of soluble fiber per 3g of total dietary fiber).
3. After treatment completion, maintain probiotic implementation with fermented foods, such as yogurt.

### Perspectives

While current research is promising, further clinical trials are needed to establish the effect of prebiotics, probiotics, and synbiotics supplementation in CC. Little is known about their interaction with chemoradiotherapy and immunotherapy treatments, so, their synergistic role needs to be demonstrated. Future research should also focus on optimal probiotic strains, dosages, and treatment duration; and, research concerning the best dietary intervention to sustain probiotic species is essential. It is also necessary to standardize formulations (i.e., synergistic synbiotics) to consider variations in individual gut microbiomes, personalized treatment approaches, and the need for predictive biomarkers of treatment response. Integrating synbiotics, probiotics, and postbiotics into CC treatment represents an exciting frontier in medical science to potentially improve patient outcomes and advance our understanding of the interplay between the gut microbiome and cancer.

### Study limitations

Even though the clinical effect of prebiotic and probiotic supplementation is important, the evidence is limited; therefore, the strength of recommendations is also low. However, prebiotics, probiotics, and synbiotics are generally regarded as safe by WHO and FDA. The recommendation of these components and organisms is safe because no evidence of adverse effects has been reported.

## Conclusions

**Figure 1:**
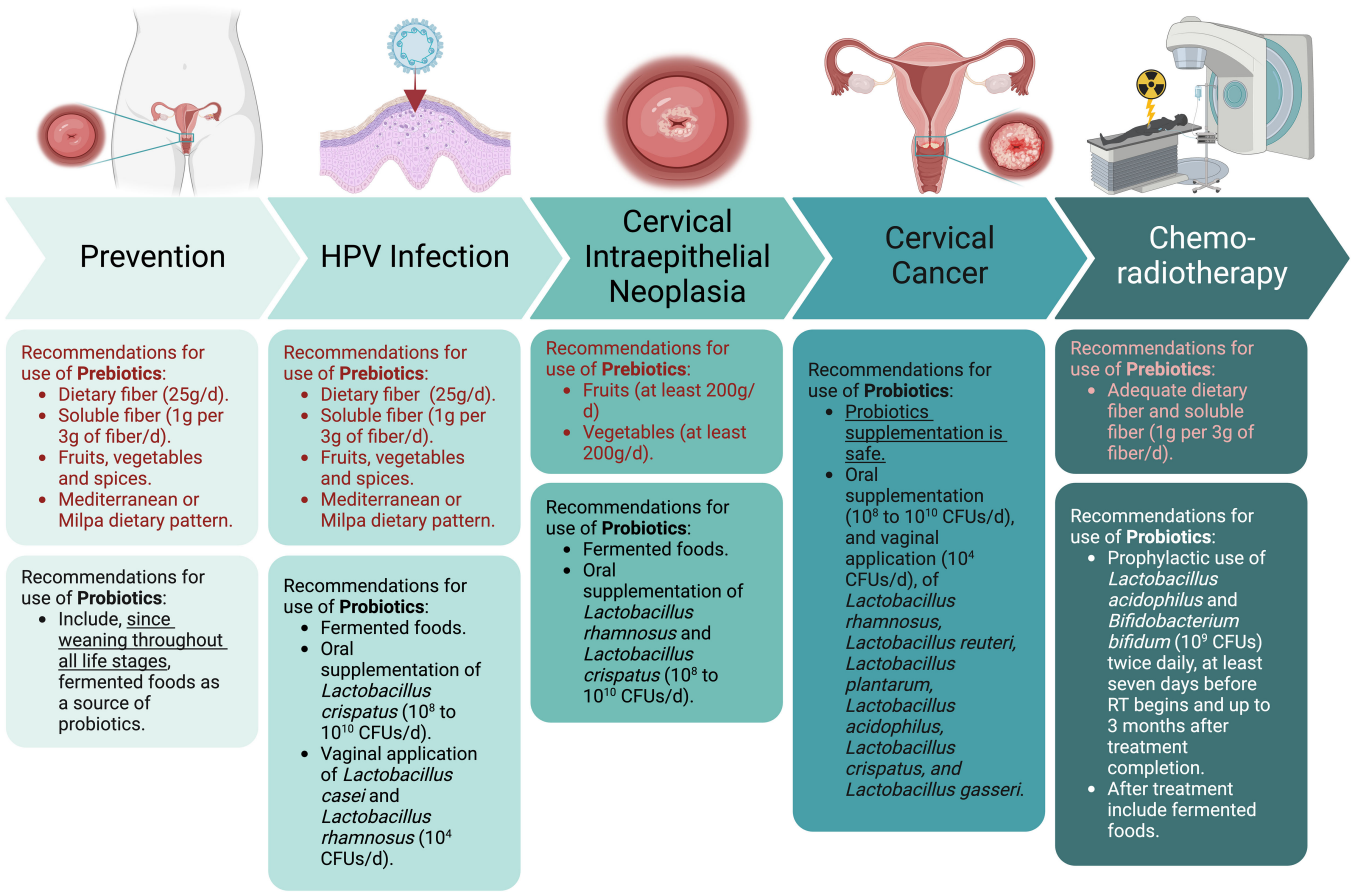
Summary of recommendations for the use of prebiotics and probiotics according to the natural evolution of cervical cancer, from prevention of HPV infection to treatment management in locally advanced cervical cancer patients. Created with BioRender.com.

This review establishes the role of prebiotics and probiotics in CC (**Figure 1**). We demonstrate that prebiotics and probiotic supplementation is a safe and novel strategy to help prevent and treat CC. The cellular and molecular mechanisms involve the antioxidant response, cell cycle arrest, apoptosis, and the antitumoral immune response. Care must be taken to promote an adequate dietary pattern that includes vegetables, fruits, and whole grains as a source of prebiotics and fermented foods as a source of probiotics throughout life.

## Author contributions

GS: Data curation, Investigation, Writing – original draft, Writing – review & editing. MG: Investigation, Writing – original draft, Formal analysis, Methodology. AE: Formal analysis, Investigation, Methodology, Writing – original draft. JI: Formal analysis, Writing – original draft. DC-E: Conceptualization, Investigation, Supervision, Validation, Visualization, Writing – original draft, Writing – review & editing.
